# When intensity of deltamethrin resistance in *Anopheles gambiae s.l.* leads to loss of Long Lasting Insecticidal Nets bio-efficacy: a case study in north Cameroon

**DOI:** 10.1186/s13071-016-1420-x

**Published:** 2016-03-08

**Authors:** Josiane Etang, Cédric Pennetier, Michael Piameu, Aziz Bouraima, Fabrice Chandre, Parfait Awono-Ambene, Coosemans Marc, Vincent Corbel

**Affiliations:** Institut de Recherche de Yaoundé (IRY), Organisation de Coordination pour la lutte contre les Endémies en Afrique Centrale (OCEAC), B.P. 288, Yaoundé, Cameroun; Faculty of Medicine and Pharmaceutical Sciences, University of Douala, P.O. Box 2701, Douala, Cameroon; Institut de Recherche pour le Développement (IRD) UMR224 MIVEGEC, 34394 Montpellier, France; Institut Pierre Richet (IPR), BP1500, Bouaké, Côte d’Ivoire; Ecole des Sciences de la Santé, Université Catholique d’Afrique Centrale, B.P. 1110, Yaoundé, Cameroun; Centre de Recherche Entomologique de Cotonou (CREC), 01 B.P. 4414, Cotonou, Bénin; Institute of Tropical Medicine, Department Parasitology - Entomology, Nationalestraat 155, B-2000 Antwerpen, Belgium; University of Antwerp, Antwerp, Belgium; Department of Entomology, Faculty of Agriculture, Kasetsart University, Bangkok, 10900 Thailand

**Keywords:** Deltamethrin, *Anopheles gambiae s.l*, Malaria, Resistance intensity, Long-Lasting Insecticidal Nets, Vector control

## Abstract

**Background:**

In Cameroon, insecticide resistance in *Anopheles* (*An.*) *gambiae s.l.* has been reported in several foci, prompting further investigations on associated patterns of Long-Lasting Insecticidal Nets (LLINs) bio-efficacy. The current study, conducted from June to August 2011, explored the intensity of deltamethrin resistance in *An. gambiae s.l.* from Pitoa and its impact on the residual bio-efficacy of LifeNet, a LLIN with deltamethrin incorporated into polypropylene nets (PND).

**Methods:**

Two-four days old females *An. gambiae s.l.* reared from larval collections in Pitoa were tested for susceptibility to DDT, permethrin and deltamethrin, using standard World Health Organization (WHO) tube assays. Intensity of deltamethrin resistance was explored using WHO tube assays, but across six working concentrations from 0.001 % to 0.5 %. Bio-efficacy of unwashed and washed PND was assessed using WHO cone test. Species identification and *kdr 1014* genotyping were performed on mosquito samples that were not exposed to insecticides, using PCR-RFLP and HOLA methods respectively. The Kisumu reference susceptible strain of *An. gambiae s.s.* was used for comparisons.

**Results:**

A total of 1895 *An. gambiae s.l.* specimens from Pitoa were used for resistance and PND bio-efficacy testing. This mosquito population was resistant to DDT, permethrin and deltamethrin, with 18–40 min knockdown times for 50 % of tested mosquitoes and 59–77 % mortality. Deltamethrin Resistance Ratio compared with the Kisumu strain was estimated at ≥500 fold. LifeNets were effective against the susceptible Kisumu (100 % knockdown (KD_60min_) and mortality) and the resistant Pitoa samples (95 % KD_60min_, 83–95 % mortality). However, the bio-efficacy gradually dropped against the Pitoa samples when nets were washed (*X*^2^ = 35.887, df = 8, *p* < 0.001), and fell under the WHO efficacy threshold (80 % mortality and/or 95 % KD_60min_) between 10 and 15 washes. The Pitoa samples were composed of three sibling species: *An. arabiensis* (132/154, 86 %), *An. coluzzii* (19/154, 12 %) and *An. gambiae s.s.* (3/154, 2 %). The *kdr L1014F* allele was found only in *An. coluzzii* (N_positive_ = 13/19), at 34 % frequency and heterozygote stage. No specimen carried the *kdr L1014S* allele.

**Conclusions:**

The current study showed that LifeNet might still offer some protection against the resistant *An. gambiae s.l.* population from Pitoa, provided appropriate dose of insecticide is available on the nets.

## Background

Insecticide resistance in malaria vectors is a growing issue that jeopardizes efforts toward malaria elimination, since the general use of insecticides in Indoor Residual Spraying (IRS) or Long-Lasting Insecticidal Nets (LLINs) constitutes the only means of mass prevention of the disease [1]. Resistance is of particular concern in sub-Saharan Africa, where DDT and pyrethroid resistance is widespread in the major malaria vector species, i.e. those of the *Anopheles* (*An.*) *gambiae* complex and the *An. funestus* group [2, 3].

In Cameroon, malaria annually accounts for 35 to 40 % of deaths in health facilities, 40 to 45 % of outpatient consultations, and 30 % of hospitalisations. It is also responsible for 26 % of job and school absenteeism and 40 % of health spending in homes [4]. Malaria control efforts have been intensified over the last five years, and vector control is highly prioritized in the national strategic plan for malaria control [5]. Over eight and half million LLINs were distributed for free to the general population in 2011, covering at least 80 % of pregnant women and children under five years old. The National Malaria Control Programme also aims to distribute twelve million LLINs in 2015 and 2016. The impact of these nationwide malaria vector control interventions on the disease burden, alongside implementation of rapid diagnostic tests and case management with Artemisinin combined therapy (ACT), is yet to be documented. Meanwhile, DDT and pyrethroid resistance is increasingly reported in three major malaria vectors belonging to the *An. gambiae* complex [6]: *An. gambiae s.s, An. coluzzii* and *An. arabiensis* [7–10]. Pyrethroid resistance in *An. gambiae s.l.* was first reported in the Pitoa health district in the northern region of Cameroon. Subsequently, it has been reported in *An. gambiae s.l.* from four more of the ten Regions (East, Centre, Littoral and West). The resistance has been conferred by two main mechanisms: (1) increased detoxification through high levels of mixed function oxidases, glutathione S-transferases or non-specific esterases and (2) alterations of insecticide target site via *kdr* mutations in the gene coding for the voltage gated sodium channel. Some mosquito populations even display multiple insecticide resistance including both *kdr* mutations and metabolic-based mechanisms [10, 11].

However, little is known about the magnitude and implications of insecticide resistance at the operational level, since many confounding factors make the interpretation of trial outcomes difficult [12]. Until recently, pyrethroid resistance based on *kdr* mutations in *An. gambiae* from Côte d’Ivoire [13, 14] did not adversely affect the effectiveness of pyrethroid-treated bed nets in terms of reduction of man-vector contact and asymptomatic infections, and in terms of protection against malaria attacks. Conversely, studies conducted in southern Benin showed a lower effectiveness of LLINs against resistant mosquitoes, with neither asymptomatic infections, nor malaria attacks, being reduced [15–17].

Considering historic and contemporary data on *An. gambiae s.l.* vector species and their resistance phenotypes, malaria burden and intervention coverage, the Pitoa health district in Cameroon appears as an ideal location for further investigations of the magnitude of insecticide resistance and its effects on conventional vector control tools. Due to the complexity of the malaria transmission system and the rapid increase of *An. gambiae s.l.* resistance to insecticides in Pitoa, achieving malaria elimination is anticipated to be difficult in this health district. Malaria incidence increased from 54.4 % in 2002 [18] to 61.5 % in 2011 [Etang and Bigoga, personal communication]. This incidence is among the highest of the twelve health districts of northern Cameroon region, including Garoua Nord and Garoua Centre health districts (around 46 % malaria incidence).

The objective of this study was to evaluate Bayer LifeNet (batch 2010–004024) under laboratory conditions and assess its intrinsic bio-efficacy and wash resistance against the pyrethroid-resistant strain of *An. gambiae s.l.* from Pitoa. For this purpose, we updated the status of DDT and pyrethroid resistance in *An. gambiae s.l.* population from Pitoa as defined by the WHO standard criteria. Dose–response tests subsequently revealed a noteworthy increase of deltamethrin resistance in this *An. gambiae s.l.* population compared with the Kisumu susceptible reference strain of *An. gambiae s.s.* After 10–15 washes, the deltamethrin on the LifeNet was no longer effective against the *An. gambiae s.l* population from Pitoa.

## Methods

### Study site

The study was conducted in the Pitoa health district (09°23′31”N, 13°30′09”E), from June to August 2011. Pitoa is small city situated at about 15 km from Garoua town, which is the capital city of the northern region of Cameroon.

The epidemiology of malaria in Pitoa is particularly complex, owing to its agro-economic and climatic environments and the variety in parasite and vector species compositions [18]. This health district is surrounded by cotton growing fields in the savannah area (about 35 000 ha cultivated area). Malaria transmission is seasonal, with a sudden rise of new infections acquired during the rainy season (May-October). There is a peak transmission season (September-October) and a low transmission season (April-May). Malaria infection is essentially due to *Plasmodium falciparum*, with few *P. malariae.* Several *Anopheles* species have been incriminated as vectors, mainly those of the *An. funestus* group and *An. gambiae* complex [18]. The dynamics of vector populations, parasite transmission and disease burden with the roll out of LLINs is currently under in-depth investigation [Etang and Bigoga, personal communication].

However, previous studies showed a moderate level of resistance to permethrin, deltamethrin and DDT in *An. gambiae s.l.,* mainly due to high oxidase and esterase activities [8, 9, 11]. Furthermore, the *kdr L1014F* mutation was reported in the local population of *An. gambiae s.s.*, although at very low frequency [19].

### Mosquitos collection and sampling

*An. gambiae s.l.* larvae and pupae were collected from breeding sites in August 2011 and reared locally until adult emergence. Adult mosquitoes were identified using morphological identification reference keys [20, 21]. Only females *An. gambiae s.l.* aged two-four days old were used for insecticide resistance and LifeNet bio-efficacy testing.

### Resistance testing

DDT and pyrethroid resistance was tested using the standard World Health Organization (WHO) susceptibility test procedures for adult mosquitoes. Tests were performed under ambient room temperature (25–28 °C) and relative humidity of 70–80 % [22].

Mosquitoes were exposed for one hour to diagnostic concentrations of DDT (4 %), deltamethrin (0.05 %) orpermethrin (0.75 %) on impregnated papers, purchased from University Sains, Malaysia. For each insecticide concentration, susceptibility bioassays were performed with five batches of 20–25 unfed females: four batches were exposed to insecticide-impregnated filter papers and one batch was exposed to untreated filter paper as a control. After one hour-long exposure, mosquitoes were transferred to holding tubes and provided with cotton pads soaked with 10 % sugar solution. The number of mosquitoes knocked-down was recorded at five minute intervals during the one hour-long exposure and mortality was determined 24 h post exposure.

Resistance status was evaluated according to the WHO criteria [22], which classify mortality rates of less than 90 % as indicative of resistance while those greater than 98 % indicate susceptibility. Mortality rates between 90–98 % suggest the possibility of resistance that needs to be verified.

Tests were concomitantly performed with the Kisumu susceptible reference strain of *An. gambiae s.s.* maintained in the OCEAC (Yaoundé, Cameroon) insectaries.

### Evaluation of resistance intensity

The intensity of deltamethrin resistance in the Pitoa wild *An. gambiae s.l.* population was also assessed using WHO standard tube test protocol for adult mosquitoes [22], with a range of 5 deltamethrin concentrations (0.001, 0.005, 0.01, 0.1 and 0.5 %), in addition to the classic diagnostic concentration (0.05 %).

Filter paper sheets were impregnated with technical-grade deltamethrin (SIGMA ALDRICH) or control solution (acetone + silicon oil) by the research team, at least 24 h prior to use. A stock solution of 1 % deltamethrin was prepared by mixing 204 mg of the commercial product (98 %) with 20 ml of acetone. Then, six working solutions of deltamethrin were prepared by serial dilution of the stock solution with acetone and silicon oil (40 ml silicon + 60 ml acetone = 100 ml dilution solution), to obtain 0.001, 0.005, 0.01, 0.1 and 0.5 % working solutions respectively. Acetone acted as the solvent; silicon oil (SIGMA ALDRICH) served as the carrier for deltamethrin. For each deltamethrin concentration, four sheets of filter paper (12×15 cm WhatmanN°1) were impregnated with two millilitres of working solution (deltamethrin + acetone + silicon oil) each, using a micropipette.

A batch of four filter paper sheets was impregnated with acetone + silicon solution for use as control. The impregnated papers were allowed to dry on a wire fence for 24 h at room temperature (27–30 °C), then were wrapped in aluminum foil and stored at 4 °C until the date of the test.

For each of the six deltamethrin concentrations, four to five batches of 20–25 mosquitoes were exposed for one hour to deltamethrin-impregnated papers. The number of mosquitoes knocked down was recorded at five minute intervals during the one hour-long exposure and mortality was determined 24 h post exposure.

Tests were concomitantly performed with same range of deltamethrin concentrations on the Pitoa wild mosquito samples and laboratory samples of the Kisumu susceptible reference strain of *An. gambiae s.s.* maintained in the OCEAC (Yaoundé, Cameroon) insectaries. The increase in tolerance or resistance level in adult mosquitoes of the Pitoa population was calculated by comparing their knock-down times and mortality with those of the Kisumu susceptible strain.

### Assessment of long lasting insecticidal nets bio-efficacy

#### Long lasting insecticidal nets and washing procedures

Long Lasting Insecticidal Nets used in this study were from LifeNet brand, a WHO interim recommended LLIN. According to the manufacturer (Bayer CropScience), the fabric is made of polypropylene and has a deltamethrin load of 8.5 a.i g/kg for 100 denier net fabric (PND), resulting in a deltamethrin concentration of 340 mg a.i./m^2^ incorporated into the fibre material.

A 30 cm × 30 cm piece of polyester netting (100 denier) treated with deltamethrin at 25 mg a.i/m^2^ (hand-dipped PRD) by the research team using the dipping method was used as positive control and untreated polyester nets as negative control.

For this study, Bayer provided samples of LifeNet wrapped in aluminium foil (washed and unwashed), one piece of 30 cm × 30 cm netting for each of 0, 5, 10, 15, 20, 25, 30 or 35 washes. LLIN samples were kept in aluminum foil and stored at room temperature (27–30 °C) until the date of the test. Details of standard washing and bioassays are provided in the WHO guidelines for testing and evaluation of LLINs [23]. Briefly, washing included placing a net sample into a 1-l beaker in which 2 g/l soap (savon de Marseille, pH 10–11) was fully dissolved into 0.5 l deionized water. The beakers were introduced into a 30 °C water-bath and shaken for 10 min at 155 movements per minute. The samples were then removed, rinsed twice for 10 min in clean, deionized water under the same shaking conditions as above, dried at room temperature and stored at 30 °C in the dark between washes.

### Cone test

Bio-efficacy of LifeNet against the Pitoa field collected and the Kisumu laboratory reared samples of *An. gambiae s.l.* was assessed using the WHO cone test to measure knock-down and mortality of mosquitoes after contact with nets [23]. Batches of five non-blood fed, two to four days old *An. gambiae s.l.* females were exposed for three minutes to net samples and held for 24 h with access to sugar solution. One hundred mosquitoes (5 mosquitoes × 20 cones) were exposed to each of the net samples and results pooled for analysis. Mosquitoes exposed to hand-dipped PRD and untreated nets were positive and negative controls, respectively. Bioassays were carried out at 27 ± 2 °C and 75 ± 10 % RH. Knock-down was measured at 60 minutes post- exposure and mortality after 24 h.

The number of washes and bioassays performed with each of the Kisumu susceptible and the Pitoa resistant strains of *An. gambiae s.l.* are summarized as followed:

- one PND (LifeNet) sample x five mosquito replicates x height configurations (0,5,10,15,20,25, 30 and 35 washes) = forty cone tests (eight hundred females);
- one PRD (hand treated with deltamethrin using the dipping method) sample x five replicates x one configuration = five cone tests (one hundred females);
- one untreated sample x five replicates x one configuration = five cone tests (one hundred females).

### Species identification and *kdr* genotyping

Mosquitoes used as control during susceptibility, resistance intensity and cone tests were used for species identification and *kdr 1014* genotyping, in order to estimate the frequencies of *kdr* alleles in the tested samples. DNA was extracted from each specimen using the method of Collins et al. [24], and each individual was identified to the species level using PCR-RFLP [25]. This method allows simultaneous identification of the species of the *An. gambiae* complex. Alleles at the *kdr1014* locus were genotyped using hot oligonucleotide ligation assay (HOLA) as described by Lynd et al. [26].

### Data analysis

The knock-down times for 50 and 95 % (KDT_50_ and KDT_95_) mosquitoes during exposure to insecticide impregnated papers in susceptibility tests were estimated using a log-time probit model [27]. The log-probit analysis was performed using the WIN DL (version 2.0, 1999) software. The KDT_50_ recorded from field-collected mosquitoes were compared with that of the Kisumu reference susceptible strain of *An. gambiae* by estimates of KDT_50_ Ratios (KDT_50_R).

The minimum plausible Resistance Ratio (RR) was estimated based on lethal concentration for 100 % mosquitoes of the Pitoa samples and that of the Kisumu susceptible samples as followed: RR = LC_100_ Pitoa/LC_100_ Kisumu.

Bio-efficacy of mosquito nets was estimated by means of mosquito knock-down rates 60 min post exposure to treated-nets (KD_60min_) and mortality rates 24 h post exposure via cone test. The number of washes generating mortality and/or KD_60min_ above the cut-off point (more than 80 % mortality after 24 h and/or above 95 % KD_60min_) was reported for each strain. The variations of KD_60min_ and mortality rates in the Pitoa *An. gambiae s.l* population in contact with washed LLINs were analysed using the Pearson’s Chi-square test of independence, with R software (Version 2.15.2, R Development Core Team 2005).

## Results

### Status of DDT and pyrethroid resistance

A total of 314 *An. gambiae s.l.* females from Pitoa were used for susceptibility tests: 294 exposed to insecticides (4 % DDT, 0.75 % permethrin or 0.05 % deltamethrin) and 20 control. Also, 278 *An. gambiae s.s.* females from the Kisumu strain were tested: 258 exposed to insecticides and 20 control. The number of mosquitoes exposed to a given diagnostic concentration of insecticide varied from 85 to 110. The recorded knock-down (KD) times and mortality rates are given in Table 1.

**Table 1:** Knock-down times for 50 and 95 % of the Kisumu susceptible strain and the Pitoa *Anopheles gambiae s.l.* population to DDT, permethrin and deltamethrin diagnostic concentrations

Diagnostic concentrations	Kisumu strain				Pitoa popualtion				
	N	kdT_50_[CI] (min)	kdT_95_[CI] (min)	Mortality (%)	N	kdT_50_[CI] (min)	kdT_95_[CI] (min)	KDT_50_R	Mortality (%)
4 % DDT	80	19.8 [17.5–21.3]	31.2 [28.5–33.1]	100	85	40.3 [38.1–43.1]	>60	2.0	76.5
0.05 % Deltamethrin	91	10.1 [8.2–12.8]	22.2 [20.1–24.9]	100	110	19.6 [17.9–21.3]	37.6 [33.2–39.5]	1.9	59.1
0.75 % Permethrin	87	9.1 [7.5–11.5]	21.4 [19.2–23.3]	100	99	21.9 [18.7–23.5]	45.3 [39.8–48.5]	2.4	72.7

*N* Sample size, *CI* confidence interval at 95 %, *kdT*_*50*_ Knock-down times for 50 % of exposed mosquitoes, *KDT*_*95*_ Knock-down times for 95 % of exposed mosquitoes, *KDT*_*50*_ *R* Ratio KDT_50_Pitoa/KDT_50_ Kisumu, *min* time in minutes

Knock-down and mortality rates were 0–3 % in control mosquitoes from either the Kisumu strain or the Pitoa wild population.

The Kisumu strain was fully susceptible to the three insecticides. The knock-down times for 50 % (KDT_50_) mosquitoes were 20 min in contact with DDT and around 10 min with permethrin or deltamethrin. For the three insecticides, KDT_95_ were less than 32 min and 100 % exposed mosquitoes were dead at 24 h post exposure.

Conversely, the Pitoa population of *An. gambiae s.l.* was found to be resistant to the three insecticides. The KDT_50_ was around 40, 20 and 22 min with DDT, deltamethrin and permethrin respectively. The KDT_50_ Ratio compared to the Kisumu strain ranged from 1.9 to 2.4 fold. At the end of the 60-min exposure time, knock-down rate did not reach 95 % for DDT. Meanwhile, the KDT_95_ were around 38 and 45 min for deltamethrin and permethrin respectively. For the three insecticides, the mortality rates at 24 h ranged from 59 % to 77 %.

### Resistance intensity

A total of 575 females *An. gambiae s.l.* from Pitoa were used for resistance intensity test, including 514 specimens exposed to a range of 6 deltamethrin concentrations (0.001 % to 0.5 %) and 61 specimens used as control. Also, 585 females *An. gambiae s.s.* from the Kisumu strain were tested, including 520 specimens exposed to deltamethrin impregnated papers and 65 control specimens. The number of mosquitoes submitted to each one of the six deltamethrin concentration varied from 70 to 110, depending on the number of larvae previously collected from the field and availability of two-four days females on the day of the test.

Resistance intensity was expressed as a dose–response effect in terms of KDT_50_, KDT_95_ and mortality rates to deltamethrin (Table 2). Knock-down and mortality rates of control mosquitoes ranged from 0 % to 4 % in both the Kisumu strain and the Pitoa population.

**Table 2:** Knock-down times for 50 and 95 % of the Kisumu susceptible and the Pitoa resistant *Anopheles gambiae s.l.* strain to increased concentrations of deltamethrin on impregnated papers

Deltamethrin dosage (%)	Kisumu strain			Pitoa strain			
	N	kdT_50_[CI] (min)	kdT_95_[CI] (min)	N	kdT_50_[CI (min)]	kdT_95_[CI] (min)	KdT_50_ ratio
0.001	80	34.6 [12.2–42.1]	>60	78	>60	>60	2.2
0.005	96	18.6 [17.9–19.4]	26.1 [24.4–28.9]	67	48.3 [40.7–60.2]	>60	2.5
0.01	68	18.4 [17.5–19.4]	27.2 [24.9–31.0]	98	21.7 [18.5–24.9]	57.2 [45.8–82.3]	1.2
0.05	90	3.9 [5.9×10^–6^–2.6×10^6^]	17.7 [9.1×10^−3^–3.4×10^4^]	110	17.8 [15.9–19.6)	59.3 [52.0–69.9]	4.6
0.1	92	1.3 [7.5×10^−5^–2.6×10^4^]	9.3 [0.06–1.4×10^3^]	74	9.6 [8.6–10.0]	32.3 [28.0–38.5]	7.4
0.5	94	0.8 [1.8×10^−8^–3.7×10^7^]	6.6 [2.5×10^−4^–1.6×10^5^]	87	7.0 [4.0–9.0]	17.2 [14.1–24.3]	8.7

*N* Sample size, *CI* confidence interval at 95 %, *kdT50* Knockdown times for 50 % of exposed mosquitoes, *kdT*_*95*_ Knockdown times for 95 % of exposed mosquitoes, *min* time in minutes

### Times of knock-down

The KDT_50_ of the Kisumu strain decreased when the deltamethrin concentrations increased, ranging from 34.6 min at 0.001 % to 0.8 min at 0.5 %. The KDT_95_ also decreased from 65.6 min to 6.6 min. Between 0.005 and 0.01 % deltamethrin concentrations, however, the knock-down time did not decrease (≈18 min KDT_50_, 26–27 min KDT_95_).

With the Pitoa field collected mosquitoes, the KDT_50_ decreased from > 60 min at 0.001 % deltamethrin to 7.0 min at 0.5 % deltamethrin. The KDT_95_also decreased from > 60 min to 17.2 min.

The KDT_50_ Ratio (KDT_50_R) correspondingly increased from 2.2 to 8.7 when deltamethrin concentration was increased.

### Mortality rates

**Fig. 1:**
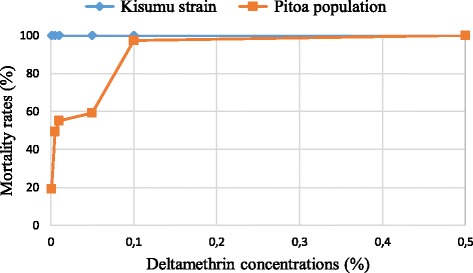
Mortality rates of Kisumu and Pitoa *Anopheles gambiae s.l.* samples in deltamethrin dose–response test

Mortality rates of Kisumu and Pitoa *An. gambiae s.l.* samples in deltamethrin dose–response tests are given in Fig. 1.

The mortality rates of the Kisumu reference strain to the 6 targeted concentrations were constantly 100 %. This maximum mortality rate did not allow estimation of the lethal concentration for 50 % mosquitoes (LC_50_) of the Kisumu strain. Conversely, mortality rates of the Pitoa population increased from 19 % to 100 %. The LC_50_ was estimated at 0.019 % deltamethrin. The concentration required to kill 100 % mosquitoes (0.5 %) was 10 fold higher than the resistance-discriminating dose for deltamethrin (0.05 %).

Although the LC_50_ of the Kisumu strain could not be estimated, its value was expected to be less than 0.001 %, i.e. the lowest deltamethrin concentration tested. Therefore, the Resistance Ratio (RR) of the Pitoa population was estimated higher than 500-fold.

### Bio-efficacy of LifeNet

The bio-efficacy of LifeNet (deltamethrin incorporated) was evaluated before (0x) and after an increasing number of washes: 5x, 10x, 15x, 20x, 25x, 30x, 35x. Approximately 100 mosquitoes were used to test each net type.

**Fig. 2:**
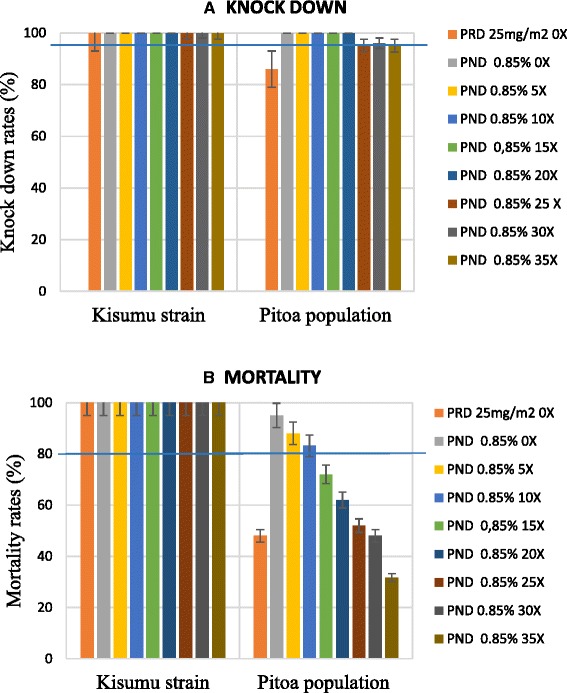
Knock-down and mortality rates of Kisumu and Pitoa *Anopheles gambiae s.l.* samples to deltamethrin treated nets. **a**: knock-down rates of *An. gambiae s.l.* samples 60 min post exposure to insecticide treated nets; **b**: mortality rates of *An. gambiae s.l.* samples 24 h post exposure to insecticide treated nets; PRD: polyester nets impregnated with deltamethrin; PND: polypropylene nets incorporating delatmethrin; Blue lines indicate cut-off point of nets efficacy (95 % knock-down rate 60 min post exposure and/or 80 % mortality rate 24 hours post exposure)

A total of 1006 females *An. gambiae s.l.* from Pitoa were used for cone tests, including 904 specimens exposed to unwashed and washed LifeNet (PND), 100 specimens exposed to positive control net (hand-dipped PRD) and 102 specimens exposed to negative control net (untreated net). Also, a total of 1006 females *An. gambiae s.l.* from the Kisumu strain were used, including 904 specimens exposed to unwashed and washed LifeNet, 101 exposed to positive control (PRD) and 100 exposed to negative control net. The recorded knock-down and mortality rates are given in Fig. 2.

With untreated net, the knockdown and mortality rates of both susceptible (Kisumu strain) and field-derived, resistant mosquito samples (Pitoa strain) were 0 %, confirming that there was no contamination during the experiments.

The hand-dipped PRD nets generated 100 % KD_60min_ and mortality against the Kisumu strain, versus 86 % KD_60min_ and 48 % mortality rates against the Pitoa strain, which corroborates Pitoa strain’s deltamethrin resistance identified in the dose–response assay.

Unwashed and washed LifeNet generated 100 % KD_60min_ and mortality rates against the Kisumu strain. Against the Pitoa strain, the KD_60min_ was mostly higher than 95 %, even after 35 washes. The subsequent mortality rates were high with unwashed nets, as well as nets washed up to 10 times (83–95 %). Nevertheless, the number of washes had a significant influence on mosquito mortality rates (*X*^2^ = 35.887, df = 8, *p* < 0.001), which gradually decreased from 95 % with unwashed PRD to 32 % with PRD washed 35 times. After 15 washes, the mortality rate dropped at 72 %, i.e. below the cut-off of LLINs efficacy.

### Species diversity and allelic frequencies at the *kdr* 1014 locus

A total of 183 *An. gambiae s.l.* specimens from Pitoa used as control (i.e. not exposed to insecticides) during susceptibility, resistance intensity and cone tests were submitted to species identification and *kdr L1014F* and *L1014S* genotyping; 154 specimens were successfully analysed. Results are given in Table 3.

**Table 3:** Species diversity and *kdr 1014* genotypes in *Anopheles gambiae s.l.* from Pitoa

Species	N (%)	*Kdr* genotypes		
		1014 L (S)	1014 F (R_w_/S)	1014S (R_e_)
*An. coluzzii* ^a^	19 (12)	6	13	0
*An. gambiae*	3 (2)	3	0	0
*An. arabiensis*	132 (86)	132	0	0
TOTAL^b^	154	141	13	-

*N* sample size, ^a^Frequency of *kdr 1014 F* allele in *An. coluzzii* = 0.34, ^b^Frequency of *kdr 1014 F* allele in overall *An. gambiae s.l.* = 0.042

Three sibling species of the *An.gambiae* complex were identified in the Pitoa population. *An. arabiensis* was the predominant species, representing 86 %, followed by *An. coluzzii* (12 %) and *An. gambiae* s.s. (2 %). The *kdr L1014F* was found only in *An. coluzzii,* at 34 % frequency, and only in theheterozygous state. The frequency of *kdr L1014F* allele in *An. gambiae s.l.* overall was 4.2 %. The *kdr L1014S *allele was not found in any of the tested specimens.

## Discussion

In this study, we attempted to address the question of possible change in the biological activity of LLINs against the *An. gambiae s.l.* population from Pitoa in North Cameroon, which is known to be resistant to DDT and pyrethroid insecticides. The findings revealed that, under laboratory testing conditions, newly operating LifeNet LLINs were effective against the local deltamethrin resistant *An. gambiae s.l.* population; but this bio-efficacy declined after nets were washed more than 10 times. By contrast, unwashed nets and nets washed up to 35 times were found effective against the Kisumu susceptible laboratory strain of *An. gambiae s.s.* The bio-efficacy of LifeNet against the Kisumu strain was consistent with the WHOPES criteria [23], in which “a LLIN would be expected to retain its biological activity for at least 20 standard washes under laboratory conditions” (>80 % mortality and/or >95 % knock-down). The intensity of deltamethrin resistance in field samples of *An. gambiae s.l.* likely played a major role in the gradual decrease of LifeNet bio-efficacy after serial washing, emphasizing the importance of assessing the magnitude of insecticide resistance and subsequent impact on vector control efficacy.

Until recently, insecticide resistance testing has been focused on discriminating doses of insecticides using either WHO tube Assay or CDC Bottle Assay. Based on these Assays, insecticide resistance in malaria vectors has been identified in at least 64 malaria-endemic countries worldwide, including 27 African countries, where it has been observed in major vector species of the *An. gambiae* complex (*An. arabiensis*, *An. gambiae* s.s. and *An. coluzzii)* and *An. funestus* group [28–30]. The resistance has been mainly linked to the presence of either *kdr* mutations (*L1024F* or *L1014S* alleles) or/and increased oxidase, esterase and glutathione S-transferase activities [31, 32]. It is noteworthy to mention that phenotypic bioassays are the definitive measure for resistance, though the detection of underlying mechanisms is also essential.

WHO tube assays performed during the current study revealed a remarkable reduction of mortality (59–76.5 %) and a moderate increase in knock-down times (less than 2.5-fold) to DDT and pyrethroids in Pitoa. In other words, the significant decrease of mortality was not associated with a radical increase in knock-down times; this pattern of pyretroid resistance in *An. gambiae s.l.* from Pitoa was previously reported by Etang et al. [8]. Therefore, variations of knock-down times appear not to be a strong phenotypic indicator of DDT and pyrethroid resistance in Pitoa, when assessed by means of diagnostic concentrations. Such resistance pattern is likely specific to metabolic-based mechanisms of resistance in *An. gambiae s.l.* [8] rather than the *kdrL1014* mutations which usually result in both high increase of knock-down times and matching decrease of mortality rates [33]. Although the *kdr L1014F* allele was recorded in the tested population of *An. gambiae s.l.* from Pitoa, the overall frequency was low (less than 5 %). This was the first report of the *kdr* mutation in *An. coluzzii* from Pitoa, which is expected to contribute to resistance in addition to previously reported metabolic detoxification, suggesting multiple resistance profiles among the three sibling species. Chouaibou et al. [19] reported both *kdr L1014F* and *L1014S* alleles at very low frequencies (1/45, 1.1 %) in *An. gambiae s.s.* specimens that survived insecticide exposure through WHO tube Assay with pyrethroids. From that time until 2011, no *kdr L1014* mutation has been reported in *An. arabiensis* from Pitoa.

The level of DDT and pyrethroid resistance recorded in the current study based on discriminative dosage and the overall *kdr L1014F* frequency is classified as moderate, according to the stratification of Strode et al. (25–80 % mortality rate and < 25 % *kdr* frequency) [34]. However, the dose response Assays with a range of deltamethrin concentrations revealed ≥500 fold magnitude of resistance in the *An. gambiae s.l*. population from Pitoa, compared with the Kisumu susceptible *An. gambiae s.s.* strain. It has been demonstrated that “intensity Assays” using different multipliers of the discriminating dose that measure the strength of resistance, rather than Assays with discriminating doses, illustrates well the resistance selection and correlate better with control failure [35].

Considering the fact that pyrethroid insecticides have been commonly used in the Pitoa cotton areas, larvae of *An. gambiae s.l.* might be exposed to selection pressure in their breeding sites. Furthermore, several other factors might influence the intensity of selection and the development of resistance in the Pitoa, including the number of genes interacting to produce the resistant phenotype, the dominance relationship of the alleles as well as the size and proportion of the population affected by insecticide treatments as suggested by Chareonviriyaphap et al. [36]. Therefore, the observed variation in the frequencies of *kdr L1014F* allele among adults of *An. coluzzii*, *An. gambiae s.s.*, *An. arabiensis* might result from physiological or behavioural differences that cause *An. gambiae s.s* and *An. coluzzii* adults to be more extensively exposed to selective pressure from pyrethroids in LLINs and IRS, resulting in an increase of *kdr L1014F* allelic frequency than *An. arabiensis*. Indeed, *An. arabiensis* equally bites humans or animals depending on availability of the host [37]. In areas with universal coverage of LLINs or IRS, the high plasticity of biting habit of *An. arabiensis* between human and alternative hosts might reduce the frequency of its contact with treated substrates and therefore exposure to insecticide selection pressure. Meanwhile, *An. gambiae s.s.* and *An. coluzzii*, which are more anthropophagic vectors [37], would mostly be exposed to contact with and selection by insecticide treated substrates when struggling to have human blood meals in the face of insecticide treatment.

In terms of operational implications of insecticide resistance, it is likely that *kdr*-based resistance exerts a differential impact on LLIN effectiveness compared to metabolic-based resistance as suggested by Strode et al. [34]. Metabolic-based resistance has been directly implicated in operational control failure of pyrethroids against *An. funestus* from South Africa [30]. In Pitoa, a set of constitutively over-expressed antioxidant genes and a single P450, *CYP4G16*, were associated with increased tolerance to deltamethrin in the *An. arabiensis* field population. These antioxidant genes include the superoxide dismutases *SOD2* and *SOD3*, the glutathione S-transferase *GSTS1* and the thioredoxin-dependent peroxidase *TPX4* [32].

Indeed, metabolic-based resistance is likely the main DDT and pyrethroid resistance mechanisms in *An. gambiae s.l.* from Pitoa, since the *kdr L1014F* allele, which is recessive, was found at low frequency and heterozygous state in the current study. *Kdr* is considered a relatively weak form of resistance compared to metabolic-based resistance, and it is usually only when *kdr* occurs along with metabolic resistance that control fails [14, 30]. Nevertheless, the increase of *kdr* frequency in addition to previously reported metabolic-based resistance and the remarkable resistance intensity recorded from the current study call attention to the risk of subsequent failure of conventional vector control interventions in Pitoa. Although new LifeNets were effective in killing this multiple resistant *An. gambiae s.l.* population, the subsequent decrease of bio-efficacy after nets were washed emphasizes the need for further investigations on the evolution of deltamethrin resistance in Pitoa during the coming years and the associated profile of LLINs bio-efficacy. Trials of new generations of LLINs which are combined with synergists (e.g. Piperonylbutoxide) to enhance their bio-efficacy against metabolic-based resistant mosquito populations should be encouraged in the Pitoa health district and other settings with a similar insecticide resistance pattern.

## Conclusions

Data from the current study confirmed DDT and pyrethroid resistance in the *An. gambiae s.l.* population from Pitoa as previously revealed by the WHO standard susceptibility tests. Furthermore, the dose response test demonstrated a noteworthy magnitude of deltamethrin resistance in this vector population (i.e. RR ≥ 500 fold increase), compared with the Kisumu susceptible reference strain. More interestingly, newly opened LifeNet LLINs were effective against this deltamethrin resistant mosquito population, although the residual bio-efficacy declined and fewer than 80 % of mosquitoes were killed between 10 and 15 washes. The intensity of insecticide resistance and its impact on LLINs efficacy should therefore be regularly monitored in order to anticipate the replacement periods of LLINs or to choose appropriate LLINs in resistance areas.

## Abbreviations

DDT: dichloro-diphenyl-trichlorethane
DNA: deoxyribonucleic acid
HOLA: hot ligation oligonucleotide assay
KD_60min_: knock-down rate 60 minutes post exposure to insecticide treated net
*kdr*: knock-down resistance
KDT_50_: time of knock-down for 50 % tested specimens
KDT_50_R: ratio times of knock-down for 50 % between tested population and the Kisumu susceptible strain
KDT_95_: time of knock-down for 95 % tested specimens
LC_100_: lethal concentration for 100 % specimens
LC_50_: lethal concentration for 50 % specimens
LLINs: Long Lasting Insecticidal Nets
OCEAC: Organisation de Coordination pour la lutte contre les Endémies en Afrique Centrale
PCR-RFLP: polymerase chain reaction-restricted fragment length polymorphism
PND: polypropylene net treated with deltamethrin
PRD: polyester net treated with deltamethrin
RR: ratio of mortality rates between tested population and the Kisumu susceptible strain
WHO: World Health Organization
